# Natural history of castration-resistant prostate cancer in sub-Saharan African black men: a single-centre study of Nigerian men

**DOI:** 10.3332/ecancer.2018.797

**Published:** 2018-01-16

**Authors:** Jibril O Bello

**Affiliations:** Department of Surgery, Urology Unit, University of Ilorin Teaching Hospital, Ilorin 240001, Nigeria

**Keywords:** castration-resistant prostate cancer, survival, sub-Saharan Africa, black men, docetaxel

## Abstract

**Purpose:**

Native sub-Saharan African black men (SSBM) are disproportionately impacted by higher stage and incurable forms of prostate cancer (PCa). This study evaluates the natural history and survival of a cohort of SSBM with castration-resistant prostate cancer (CRPC).

**Methods:**

A retrospective study of patients with CRPC as defined by the Prostate Cancer Working Group 2 managed at a centre in sub-Saharan Africa between January 2011 and December 2015 was conducted. The principal endpoint was overall survival (OS). Potential prognostic variables were evaluated using Cox proportional hazard regression models.

**Results:**

A total of 48 patients were identified. Median (IQR) age and prostate-specific antigen (PSA) at diagnosis were 70 (64–74.5) years and 42 (8.0–123.6) ng/mL, respectively. Only 15 (31.3%) patients received docetaxel and one patient each received the novel drugs enzalutamide and abiraterone. Twenty-eight patients (58.3%) died during follow-up with a median OS of 11 (95% CI: 8–14) months. Docetaxel chemotherapy and ECOG performance status were found to be prognostic (docetaxel use: hazard ratio 0·25, 95% CI 0·10–0·67, p = 0·006; ECOG 0–2: 0·26, 0·11–0·62, p = 0·003).

**Conclusion:**

This study of SSBM with CRPC revealed a mainly unmodulated clinical course with poor access to active treatments and poor survival. Improving access to new active therapies would improve survival.

## Introduction

Compared to men from other world regions, sub-Saharan African black men (SSBM) are disproportionately impacted by higher stage and incurable forms of prostate cancer (PCa) [1–4]. This situation is probably due to the weak health system in the region and possibly a genetic predisposition to aggressive forms of PCa [3–5]. Additionally, the perennial socioeconomic challenges of the region pose more hurdles for the optimal management of these patients with advanced cancer. The late stages of PCa, though it initially responds to treatment with androgen deprivation therapy (ADT), invariably progresses to castration-resistant prostate cancer (CRPC), which has a dismal prognosis and poor survival [6, 7]. With greater proportions of SSBM being diagnosed with locally advanced or metastatic disease, the burden and impacts of the CRPC would be greater in this population. Up until 2004, only palliative treatments were available for CRPC worldwide [8]; however, in the ensuing decade, there has been the introduction of an increasing stable of active agents for clinical use [8]. Some of the active therapies approved for clinical use and included in several guidelines have proven beneficial in improving symptoms, quality of life and survival [8–11]. Unfortunately, many of these active therapies are unavailable to impacted SSBM populations due to high costs, low health insurance coverage or outright unavailability in the region. These men have not benefitted from the recent advances in CRPC care as have men in the developed world and most still receive mainly supportive care. Thus, the natural course of the CRPC may have remained largely unmodulated in the sub-Saharan Africa region. This study interrogated mature survival data of a cohort of native black men with CRPC residing in a community in Central Nigeria, western sub-Saharan Africa, to describe the natural history of the disease, evaluate overall survival (OS) and assess potential prognostic factors.

## Methods

**Figure 1.**
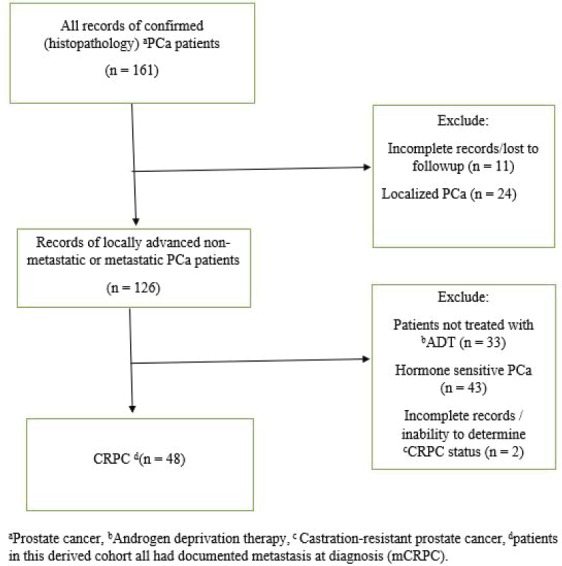
Strict selection algorithm to identify patients with CRPC.

Records of patients with pathologically confirmed PCa treated at a single public tertiary-care hospital in Nigeria, western sub-Saharan Africa, from January 2011 to December 2015 were retrieved. The hospital is typical of similar public tertiary-care centres all over the country with urology departments and a similar case load of prostate diseases, including PCa. Using a strict selection algorithm (Figure 1), patients with CRPC as defined by the Prostate Cancer Working Group 2 (relative increase of 25% and absolute increase of ≥2 ng/mL above nadir in patients on continuous ADT and castrate level of testosterone) were selected. The derived cohort was patients with CRPC and documented metastatic disease on radiographic imaging (Bone scans and radiographs, computed tomography images). Patients’ demographic data, prostate-specific antigen (PSA) level (pre-ADT PSA, nadir PSA and PSA at CRPC diagnosis), Gleason score, type of ADT, use rates of concomitant therapies (opioid analgesia, bisphosphonates, blood transfusions), symptoms prevalence (lower urinary tract symptoms [LUTS], bothersome pain, anaemia, cord compression and pathological fracture), the Eastern Cooperative Oncology Group (ECOG) performance status and survival outcomes were collected following detailed review of clinical notes. The baseline patient and disease characteristics were summarised using median and interquartile range (IQR) for continuous variables and proportions for categorical variables; patients’ ECOG performance status was categorised into two groups (Good 0–2 and Poor 3–4). The principal endpoint was OS, defined as time from diagnosis of CRPC to the date of death from any cause. Patients were censored if they were known to be alive and those lost to follow-up were not included in the analysis. Cox proportional hazards regression model was used to assess the prognostic significance of demographic and clinical variables in univariable and multivariable analyses. Kaplan–Meier plots were used to depict survival outcomes with significance determined by log rank test. All statistical analyses were performed with the SPSS version 22 software (IBM, Chicago, IL), p < 0.05 was considered significant. Institutional ethics review board approval was obtained for the study.

## Results

**Figure 2.**
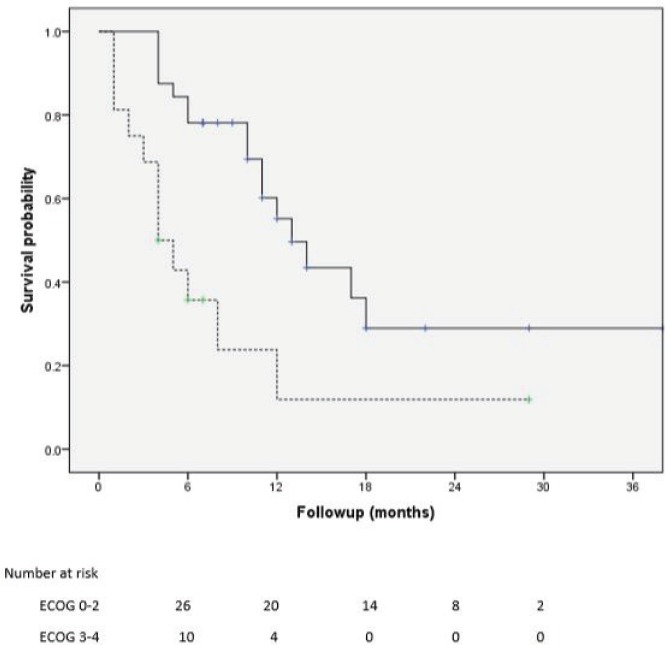
Kaplan–Meier plots of survival following CRPC diagnosis stratified by ECOG performance status (solid line: ECOG 0–2, dotted lines: ECOG 3–4); median OS is significantly different between the groups at 13 and 4 months, respectively, log rank test p = 0.007.

**Figure 3.**
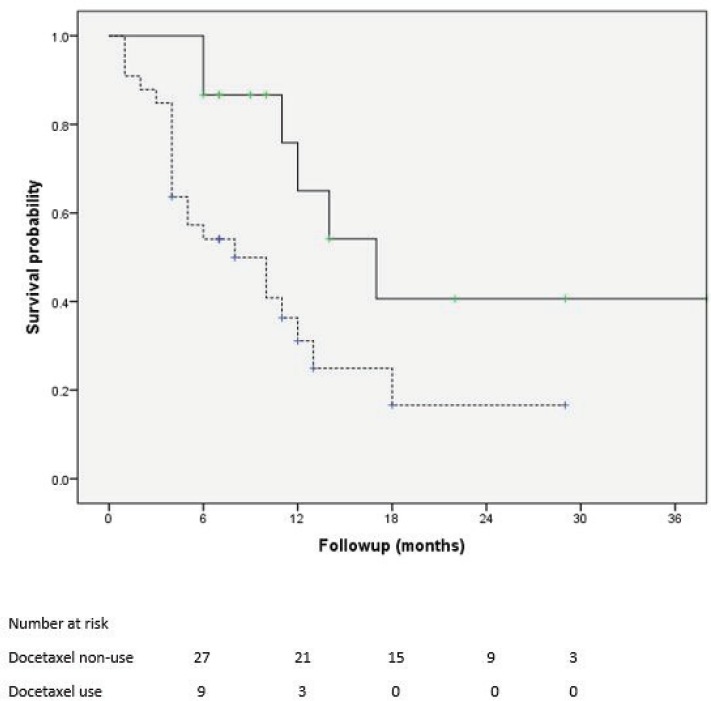
Kaplan–Meier plots of survival following CRPC diagnosis of patients who received docetaxel chemotherapy (solid line) and those who did not receive docetaxel chemotherapy (dotted line); median OS is significantly different between the groups at 17 and 8 months, respectively, log rank test p = 0.012.

Of 161 patients’ records reviewed, 48 patients diagnosed with CRPC met the inclusion criteria and were subsequently analysed. Cohort characteristics are shown in Table 1. Median age and PSA at diagnosis of CRPC were 70 years and 42 ng/mL, respectively; a quarter of the patients were resident in rural communities. Only 15 (31.3%) patients received Docetaxel (median cycles: 3, range 2–8) and 9 (18.8%) patients received the bisphosphonate – zoledronic acid (median number of doses: 3, range 2–9). A large proportion, 35 (72.9%) patients received opioids for pain and 15 (31.3%) patients received blood transfusions (median number of pints of blood: 3, range: 2–10). Of the entire cohort, only two patients (one each) received the novel drugs enzalutamide and abiraterone. A total of 28 (58.3%) patients died during a median follow-up of eight (IQR: 4–13) months and the median OS was 11 (95% CI: 7.8–14.2) months. Prognosticators of OS found on univariate analysis (Table 2) were ECOG performance status and docetaxel use status and both remained statistically significant prognosticators on multivariate analysis. Good performance status (ECOG 0–2) and docetaxel use were predictors of longer survival (HR: 0.26, 95% CI: 0.11–0.62, p = 0.003 and HR: 0.25, 95% CI: 0.10–0.67, p = 0.006, respectively). A longer OS was apparent in patients with good performance status compared to those with poor performance status (OS: 13 months, 95% CI: 9.5–16.5 versus OS: 4 months, 95% CI: 2.1–5.9; log-rank p value = 0.007) and in patients who received docetaxel chemotherapy compared to those who did not (OS: 17 months, 95% CI: 10.3–23.7 versus OS: 8 months, 95% CI: 2.6–13.4; log-rank p value = 0.003). Kaplan–Meier plots of OS by ECOG performance status and docetaxel use status (Figures 2 and 3) illustrate the differences between the survival distributions.

## Discussion

Being black confers a higher risk to developing PCa compared to other racial groups. Despite this, there has been a dearth of research into the impact of the disease on the black populations of sub-Saharan Africa. Though the better studied African-American and Afro-Caribbean men derived their ancestral origins mainly from native black populations of Africa, the wide socio-cultural and economic dissimilarities that now exists between these two groups is such that no safe assumptions of similarities in PCa clinical course or survival can be made. The few reports from Africa available in the literature reveals observations of a high prevalence of advanced disease and high mortality rates [1–4, 12]. This has been attributed to a combination of endemic poverty, inaccessible/unaffordable or unavailable curative treatment for early disease, near-absence of PSA screening, a probable genetic predisposition to clinically aggressive disease and a reduced overall life expectancy [4, 12–15].

Despite ADT, patients with advanced disease do eventually progress to CRPC. While non-metastatic CRPC is a common early clinical manifestation following castration resistance in Western men, the native African blacks in this study all had documented metastasis at CRPC diagnosis [8]. This finding may be a consequence of the differences in access to care or may reflect the lead time effect of regular PSA assay and an earlier recognition of CRPC seen in men with access to care. Frequent PSA testing or even clinic attendance is often difficult for SSBM due to cost considerations and any lead-time effect is attenuated; the men only report to clinic following the onset of bothersome symptoms due to clinical disease progression and/or new metastasis.

Surgical castration was found to be the more frequent form of ADT received by patients in this study. This may be due to its lower cost compared to medical castration. Health insurance coverage rates in sub-Saharan African region are dismally low, payments for health care are mainly an out-of-pocket expenditure and cancer treatments can easily result in financial catastrophe [16]. This situation also impacts the ability of SSBM to afford CRPC treatments due to the high costs associated with chemotherapy and novel active therapies. Only a dismal one-quarter of the men studied received first-line docetaxel chemotherapy and even less received the bisphosphonate zoledronic acid despite nine out of ten men having severe and bothersome bone pains with at least a third having skeletal-related events. Not surprisingly, only one patient each received enzalutamide and abiraterone. The abysmal use rate of the novel active therapies found in this study may be because they are still relatively new to African pharmaceutical markets having been approved for use relatively recently by the USFDA or due to the very high cost of the drugs. There are also barriers posed by culture and beliefs of the patients and practice patterns of attending physicians. Ultimately, these men unfortunately do not benefit optimally from the advancements in CRPC care.

Progression-free survival from the diagnosis of advanced disease to CRPC have been previously reported to be approximately 27 months in African men [17]; and given the natural history of advanced PCa, and the widely accepted observation that PCa-specific death commonly results from the progression of metastatic CRPC, the survival of SSBM with CRPC could be greatly impacted by the inaccessibility of active treatments. The median OS of 11 months found in this study is much lower than the demonstrated prolonged survivals in contemporary era studies of men in other regions of the world who have better access to and are receiving active treatments [10, 18–20]. This study also found that even with the demonstrated poor OS, the men who received docetaxel chemotherapy survived significantly longer than those who did not receive any docetaxel. The survival benefits derivable from the use of docetaxel chemotherapy though described over a decade ago is yet to be fully realised in sub-Saharan Africa. This finding suggests that an improved access to PCa active treatments by the underserved population of SSBM with CRPC would lead to improved survival. This could be achieved by country-wide improvements in health insurance coverage, including coverage for oncological conditions and the provision of chemotherapeutic and other drugs.

## Conclusion

Some limitations to this study to be acknowledged include the retrospective design, single-centre recruitment and a relatively small cohort; 13 patients were excluded from analysis due to missing data. Non-informative censoring was ensured by excluding patients lost to follow-up. Despite the limitations, the study is the first to describe the natural history and survival of CRPC in the high-risk but under-researched black men of sub-Saharan African origin and reveals a mainly unmodulated clinical course and poor OS.

## Figures and Tables

**Table 1.** Cohort characteristics.

Characteristics	n (%)	Median (IQR)
Age (years) Place of residence Rural Urban Pre-^a^ADT PSA (ng/mL) PSA nadir (ng/mL) PSA at ^b^CRPC (ng/mL) Biopsy Gleason score 6 3 + 4 4 + 3 8 − 10 ADT Medical castration Surgical castration ^c^ECOG performance status Good (0–2) Poor (3–4) Clinical conditions/symptoms ^d^LUTS ^e^Bothersome pains Anaemia Cord compression Pathological fracture	13 (27.1) 35 (72.9) 8 (16.7) 5 (10.4) 14 (29.2) 21 (43.7) 8 (16.7) 40 (83.3) 32 (66.7) 16 (33.3) 40 (83.3) 38 (79.2) 22 (45.8) 16 (33.3) 2 (4.2)	70 (64–74.5) 100.0 (61.8–120.8) 5.7 (1.9–24.2) 42 (8.0–123.6)

a Androgen deprivation therapy,

b castration-resistant prostate cancer,

c Eastern Cooperative Oncology Group,

d lower urinary tract symptoms,

e pains requiring continuing analgesia.

**Table 2.** Cox proportional hazards models for OS following diagnosis of CRPC.

	Univariate			Multivariate		
Variables	HR	95% CI	p	HR	95%CI	p
Age	1.02	0.96–1.07	0.569			
Rural residence	1.15	0.48–2.74	0.747			
PSA at diagnosis	1.01	0.99–1.02	0.137			
LUTS	2.15	0.70–6.57	0.181			
*ECOG (0-2)	0.36	0.17–0.80	0.012	0.26	0.11–0.62	0.003
*Docetaxel use	0.34	0.14–0.84	0.02	0.25	0.10–0.67	0.006
Biopsy Gleason score						
6	1.00 (Reference)					
7	1.26	0.45–3.54	0.663			
8-10	0.84	0.32–2.17	0.716			

* Significant at univariate analysis and multivariate analysis.
